# Supplementary material for: Validation and clinical application of a targeted next-generation sequencing gene panel for solid and hematologic malignancies
Source: PeerJ. 2020 Oct 6;8:e10069. doi: 10.7717/peerj.10069 (PMC7546223; doi:10.7717/peerj.10069)
Supplement: Supplemental Information 1 — (A) Selection of the FFPE blocks were made and 2 micron thick sections were stained with haematoxylin and eosin. A pathologist assessed the tumor cell content (TTC) and actions were completed in accordance with the % of the TCC. (B) ΔCq values. Extracted DNA was amplified in triplicate by quantitative PCR. The amount of DNA input was established by comparing the ability of DNA to be amplified in relation to a non-FFPE reference genomic DNA. A ΔCq value was calculated for each sample as follows: ΔCq = mean sample Cq value - mean non-FFPE control Cq value. A mean of ΔCq¡4was considered as appropriate for library preparation, even though values comprised between 4 < ΔCq < 6 were also suitable for library preparation. ΔCq¿6 were discarded. (D) Library quality control. Library obtained products were checked for their base pair range using a 2100 bioanalyzer instrument (Agilent, Santa Clara, CA, USA) or run in 2% agarose gel. Generated libraries in the 300-330 base pair range were considered appropriate for sequencing. [file peerj-08-10069-s001.pdf]

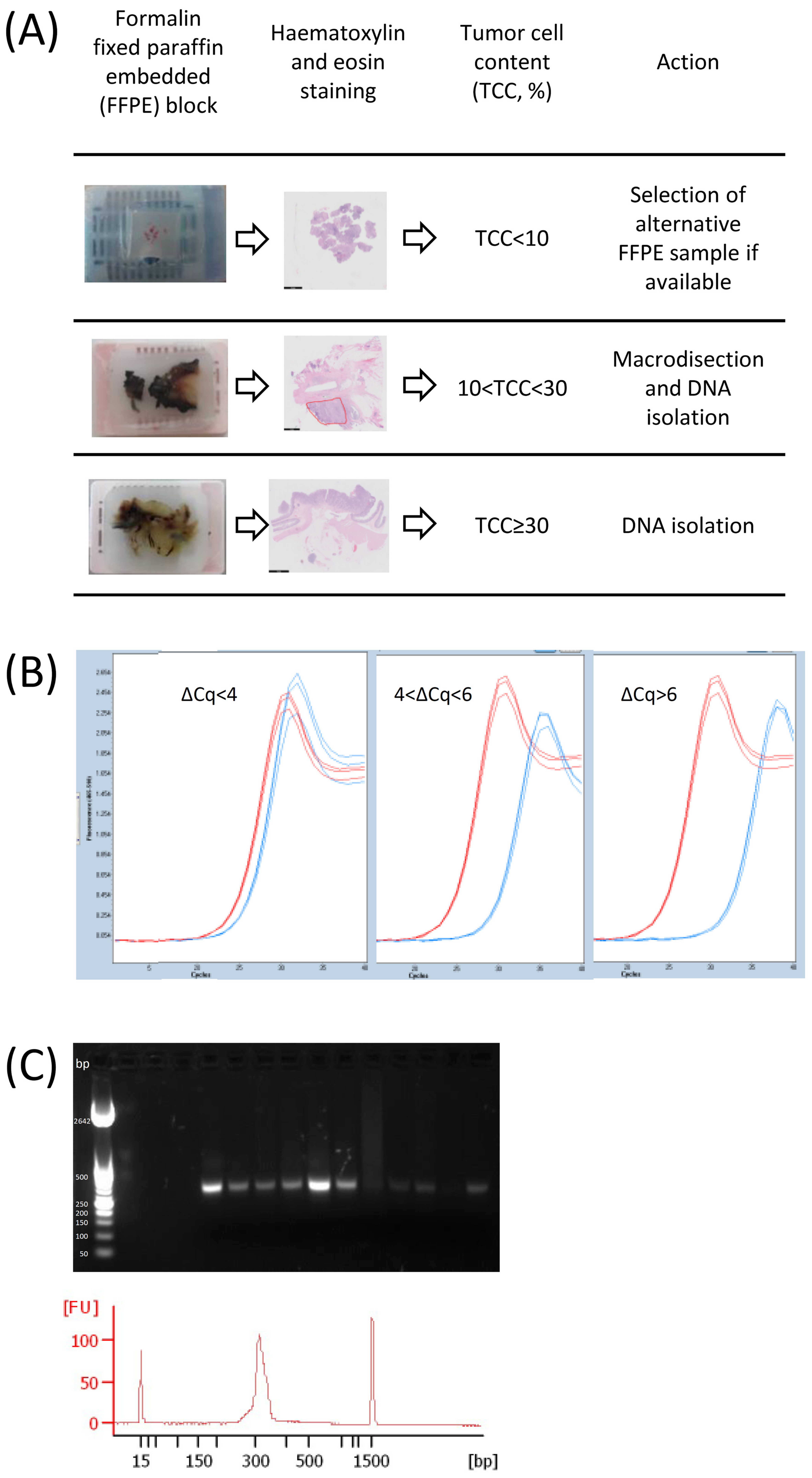
(A)
Formalin fixed paraffin embedded (FFPE) block
Haematoxylin and eosin staining
Tumor cell content (TCC, %)
Action
TCC<10
Selection of alternative FFPE sample if available
10<TCC<30
Macrodisection and DNA isolation
TCC≥30
DNA isolation
(B)
ΔCq<4
4<ΔCq<6
ΔCq>6
(C)
bp
2642
500
250
200
150
100
50
[FU]
100
50
0
15
150
300
500
1500
[bp]
