# Supplementary figures and images for: Validation and clinical application of a targeted next-generation sequencing gene panel for solid and hematologic malignancies
Source: PeerJ. 2020 Oct 6;8:e10069. doi: 10.7717/peerj.10069 (PMC7546223; doi:10.7717/peerj.10069)

(A)

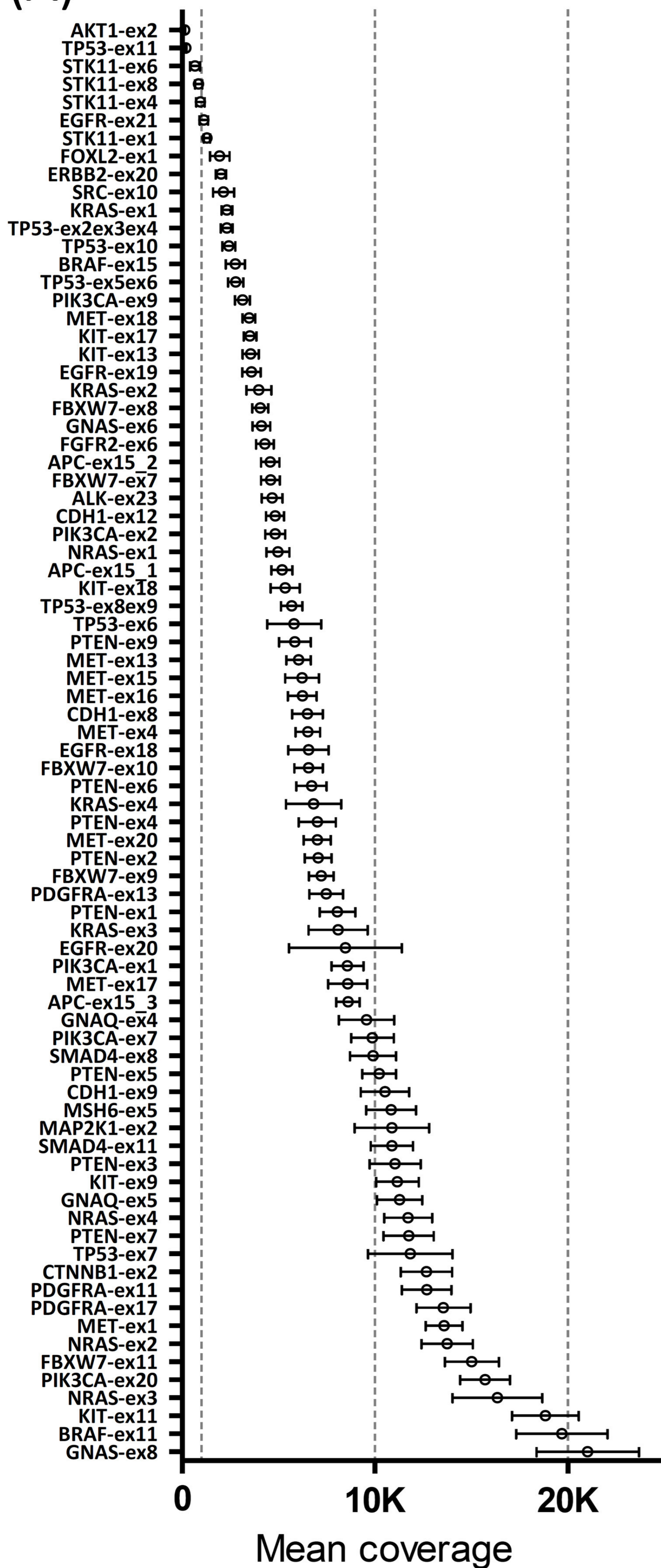

(B)

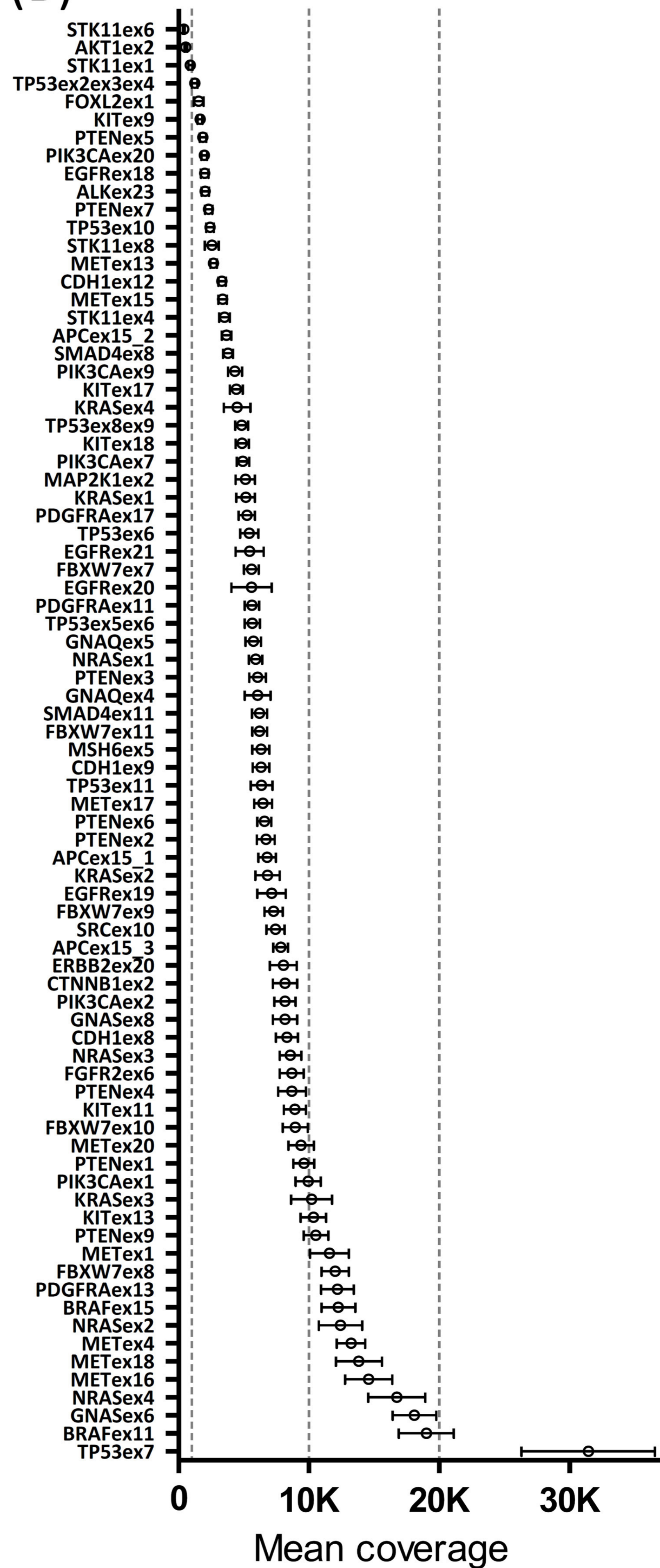

Supplement: Supplemental Information 2 — (A) pool A and (B) pool B libraries. Vertical dashed lines represent a depth of 1000x, 10000x and 20000x, respectively. Data are expressed as the mean and 95% CI. [file peerj-08-10069-s002.pdf]

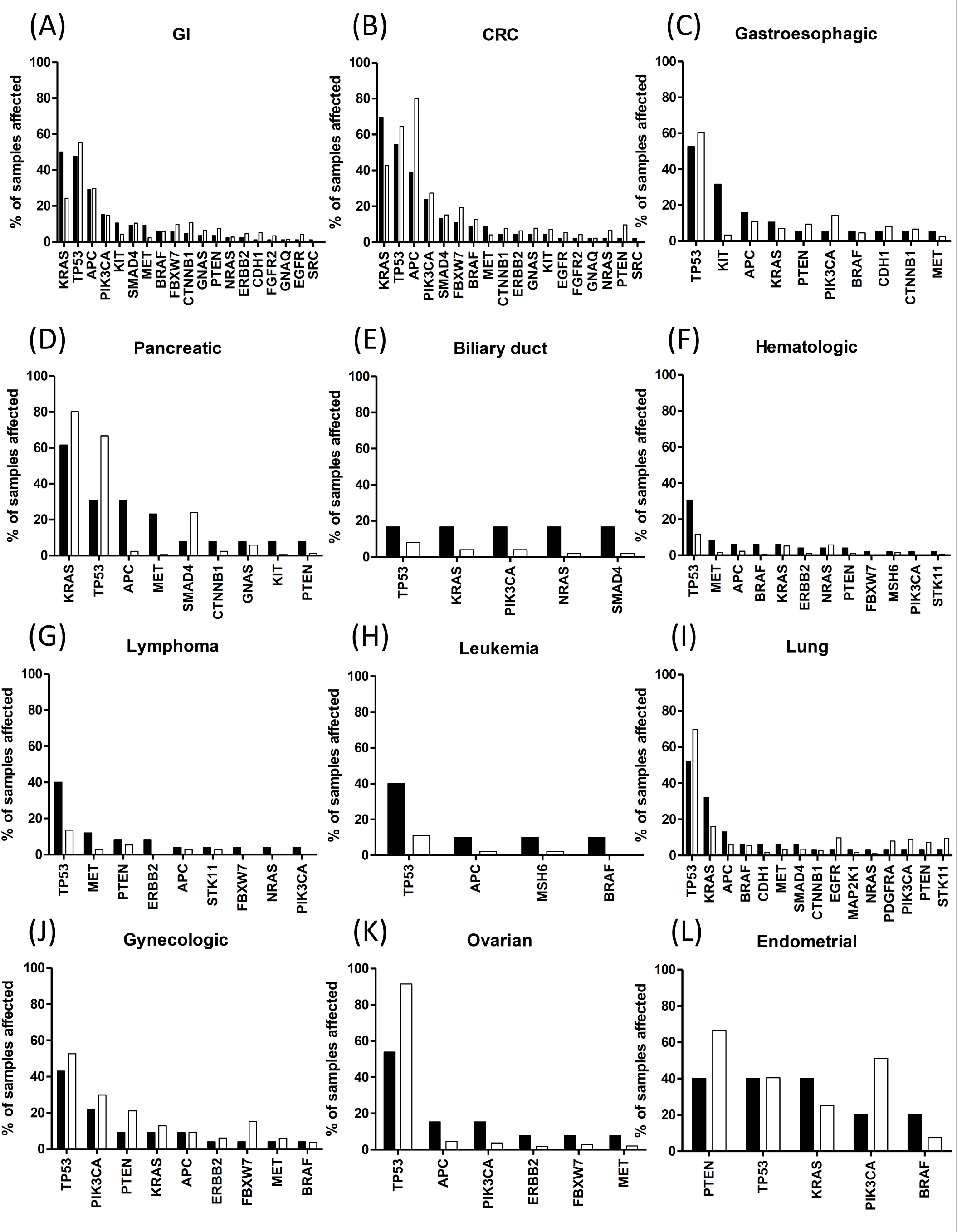

Supplement: Supplemental Information 4 — (A) Gastrointestinal (GI), n = 86 vs. n = 1737 TCGA-COAD+READ+STCA+ESCA+PAAD+CHOL+LIHC. (B) Colorectal (CRC) adenocarcinoma, n = 45 vs. n = 534TCGA − COAD + READ.(C)Gastroesophagiccarcinoma, n=19 vs.n=619 TCGA − STCA + ESCA.(D)Pancreaticcarcinoma, n=13 vs.n=171 TCGA-PAAD. (E) Biliary duct, n = 7 vs. n=50 TCGA − CHOL.(F)Hematologic, n = 49 vs. n = 173 TCGA-DLBC+LAML. (G) Lymphoma, n = 25 vs. n = 37 TCGA-DLBC. (H) Leukemia, n = 10 vs. n = 136 TCGA-LAML. (I) Lung, n = 31 vs. n = 1049 TCGA-LUAD+LUSC. (J) Gynecologic, n = 23 vs. n = 1311 TCGA-OV+CESC+UCEC+UCS. (K) Ovarian, n = 13 vs. n = 436 TCGA-OV. (L) Endometrial, n =5 vs. n = 529 TCGA-CESC. Data is expressed as percentages values of samples affected with detected variants. Black bars represent mutation frequencies resulted from the TsT26 sequencing whereas white bars correspond to values obtained from the TCGA dataset. [file peerj-08-10069-s004.pdf]

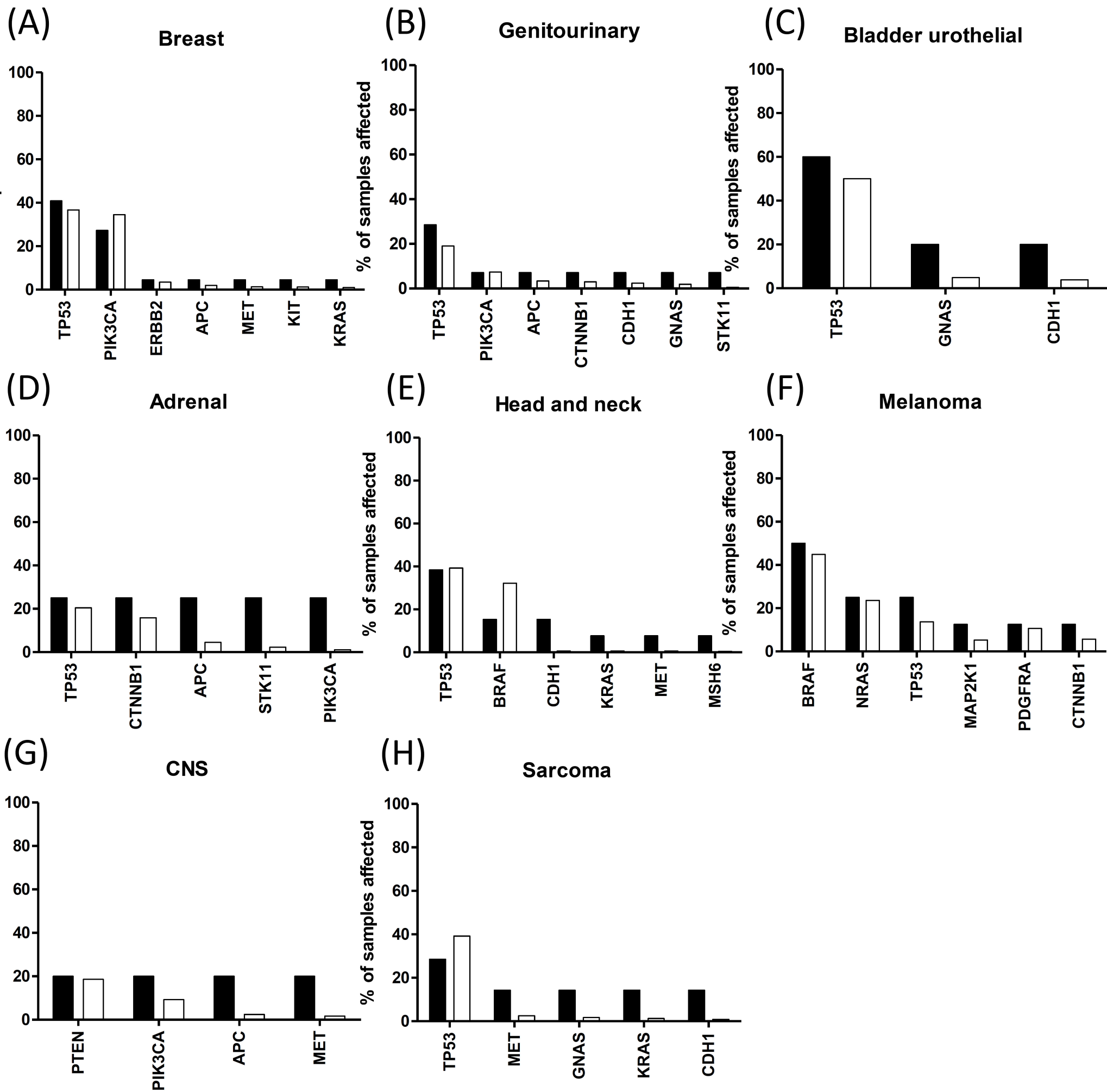

Supplement: Supplemental Information 5 — (A) Breast, n = 22 vs. n = 981 TCGA-BRCA. (B) Genitourinary, n = 14 vs. n = 1567 TCGA-BLCA+KIRC+KIRP+ACC+PRAD. (C) Bladder urothelial, n = 5 vs. n = 410 TCGA-BLCA. (D) Adrenal, n = 4 vs. n = 88 TCGA-ACC. (E) Head and neck, n = 13 vs. n = 951 TCGA-HNSC+THCA. (F) Melanoma, n = 8 vs. n = 546 TCGA-SKCM+UVM. (G) Central nervous system (CNS) n = 5 vs. n = 896 TCGA-GBM+LGG. (H) Sarcoma, n = 7 vs. n=237 TCGA-SARC. Data is expressed as percentages values of samples affected with detected variants. Black bars represent mutation frequencies resulted from the TsT26 sequencing whereas white bars correspond to values obtained from the TCGA dataset. [file peerj-08-10069-s005.pdf]
