# Supplementary material for: Validation and clinical application of a targeted next-generation sequencing gene panel for solid and hematologic malignancies
Source: PeerJ. 2020 Oct 6;8:e10069. doi: 10.7717/peerj.10069 (PMC7546223; doi:10.7717/peerj.10069)
Supplement: Supplemental Information 3 — (A) Breast, (B) Genitourinary, (C) Head and neck, (D) Melanoma, (E) Central nervous system and (F) Other solid tumor. Each column denotes an individual tumor and each row represents a gene. Detected variants are shown by grey squares whereas more than one detected variant of the same gene is depicted in black squares. [file peerj-08-10069-s003.pdf]

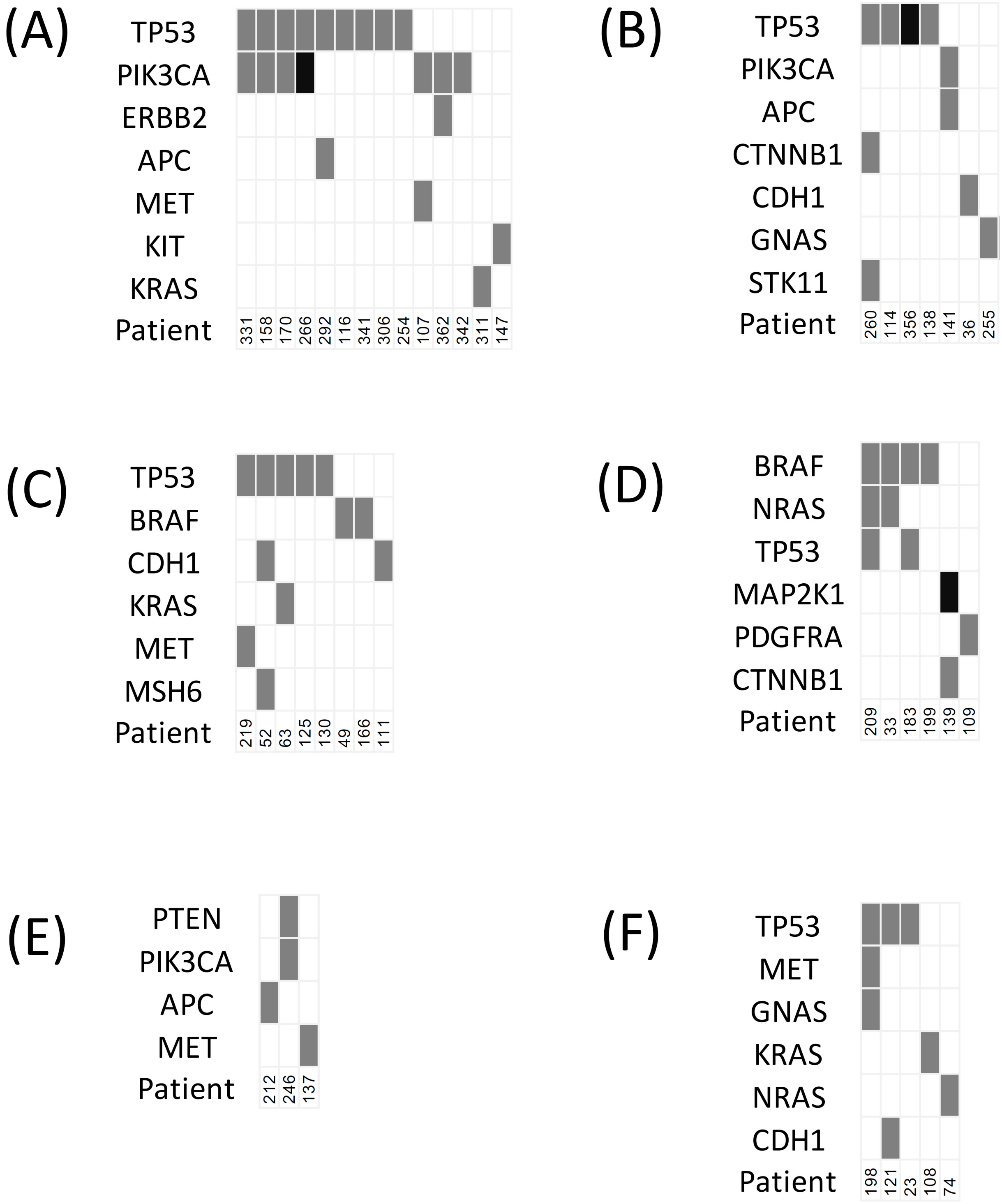
(A)
TP53
PIK3CA
ERBB2
APC
MET
KIT
KRAS
Patient
331 158 170 266 292 116 341 306 254 107 362 342 311 147
(B)
TP53
PIK3CA
APC
CTNNB1
CDH1
GNAS
STK11
Patient
260 114 356 138 141 36 255
(C)
TP53
BRAF
CDH1
KRAS
MET
MSH6
Patient
219 52 63 125 130 49 166 111
(D)
BRAF
NRAS
TP53
MAP2K1
PDGFRA
CTNNB1
Patient
209 33 183 199 139 109
(E)
PTEN
PIK3CA
APC
MET
Patient
212 246 137
(F)
TP53
MET
GNAS
KRAS
NRAS
CDH1
Patient
198 121 23 108 74
